# The genome sequence of the Riband Wave, *Idaea aversata* (Linnaeus, 1758)

**DOI:** 10.12688/wellcomeopenres.18899.1

**Published:** 2023-01-31

**Authors:** Douglas Boyes, University of Oxford and Wytham Woods Genome Acquisition Lab, Darwin Tree of Life Barcoding collective, Wellcome Sanger Institute Tree of Life programme, Wellcome Sanger Institute Scientific Operations: DNA Pipelines collective, Tree of Life Core Informatics collective, John F. Mulley, Darwin Tree of Life Consortium

**Affiliations:** 1 UK Centre for Ecology and Hydrology, Wallingford, Oxfordshire, UK; 2 School of Natural Sciences, Bangor University, Bangor, Wales, USA

**Keywords:** Idaea aversata, Riband Wave, genome sequence, chromosomal, Lepidoptera

## Abstract

We present a genome assembly from an individual male *Idaea aversata* (the Riband Wave; Arthropoda; Insecta; Lepidoptera; Geometridae). The genome sequence is 437 megabases in span. The whole assembly is scaffolded into 30 chromosomal pseudomolecules, including the assembled Z sex chromosome. The mitochondrial genome has also been assembled and is 17.5 kilobases in length. Gene annotation of this assembly on Ensembl identified 10,165 protein coding genes.

## Species taxonomy

Eukaryota; Metazoa; Ecdysozoa; Arthropoda; Hexapoda; Insecta; Pterygota; Neoptera; Endopterygota; Lepidoptera; Glossata; Ditrysia; Geometroidea; Geometridae; Sterrhinae; Idaea; Idaea aversata (NCBI:txid104447).

## Background

The Riband Wave *Idaea aversata* (Linnaeus, 1758) is a small member of the Sterrhinae subfamily of geometrid moths (wingspan up to 30 mm). It has been recorded from across the Palearctic region and throughout the UK, where it is commonly attracted to light traps from June to September. The larvae feed on low lying plants such as bedstraw, dandelion, dock, and knotgrass, and overwinter while still relatively small.

Like other members of the subfamily, the Riband Wave has wave-like patterns on the wings, and this species is readily distinguished from similar species by a distinct kink in the third (most distal) line near the leading edge of the forewing. Overall colouration is quite variable, with most individuals grey or sandy coloured, but with some red/orange examples. The species occurs in two principal recognised forms: a banded form (sometimes referred to as form *aversata*), which has a dark ribbon-like crossband running across the fore- and hindwings; and form *remutata*, which lacks the dark band and shows only the wave-like pattern of crosslines. Whilst often claimed to be present in equal numbers, Leverton found the highest frequency of the banded ( *aversata*) form to be around 30% in 2001, and only then in Kent, with a frequency nearer 15% in Yorkshire and a clear decline in frequency with higher latitude ( Leverton, 2001). In the 1950’s, Ford claimed a frequency of “about 5%” for the banded form ( Ford, 1955), and Bergmann considered the unbanded form to be “common” ( Bergmann, 1938). Breeding experiments in the 1930s and 1940s showed that a single locus determines banding, and that the banded f. *aversata* is dominant to the unbanded f. *remutata* ( Bergmann, 1938; Ford, 1953; Hawkins, 1937; Hawkins, 1952). Of the three possible genotypes, AA and Ab produce banded individuals and are generally indistinguishable from each other (although homozygotes may have a slightly darker band ( Ford, 1953), and bb individuals are unbanded. Ford (1955) suggested that there is heterozygote advantage. Other experimenters have looked at other aspects of colouration: Bergmann, who considered a reddish base colour to be recessive to the dominant grey ( Bergmann, 1938); Cockayne, who considered a pink form to be dominant to grey ( Cockayne, 1945); and Knill-Jones, who showed that an orange base colour was only found in the banded form, and that a reddish form was dominant ( Knill-Jones, 2002).

A genome assembly for the Riband Wave *I. aversata* will be invaluable to future work aimed at determining the genetic basis of colour polymorphism, and will contribute to the growing set of genomic resources for studying lepidopteran ecology and evolution.

## Genome sequence report

**Figure 1.**
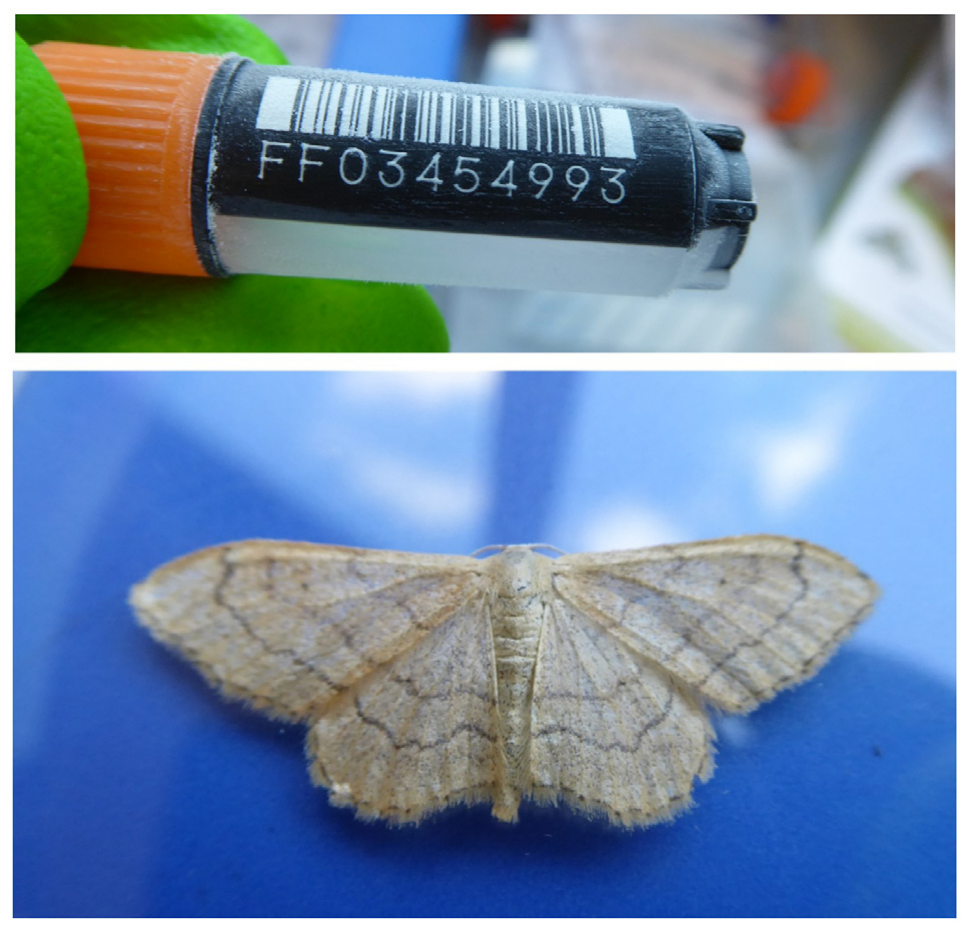
Photograph of the *Idaea aversata* (ilIdaAver1) specimen used for genome sequencing.

The genome was sequenced from one male *I. aversata* of the unbanded form ( Figure 1) collected in Wytham Woods (latitude 51.77, longitude –1.34). A total of 56-fold coverage in Pacific Biosciences single-molecule HiFi long reads and 86-fold coverage in 10X Genomics read clouds were generated. Primary assembly contigs were scaffolded with chromosome conformation Hi-C data. Manual assembly curation corrected 4 missing joins or mis-joins and removed one haplotypic duplication, reducing the assembly length by 1.7% and the scaffold number by 9.09%, and reducing the scaffold N50 by 1.4%.

**Figure 2.**
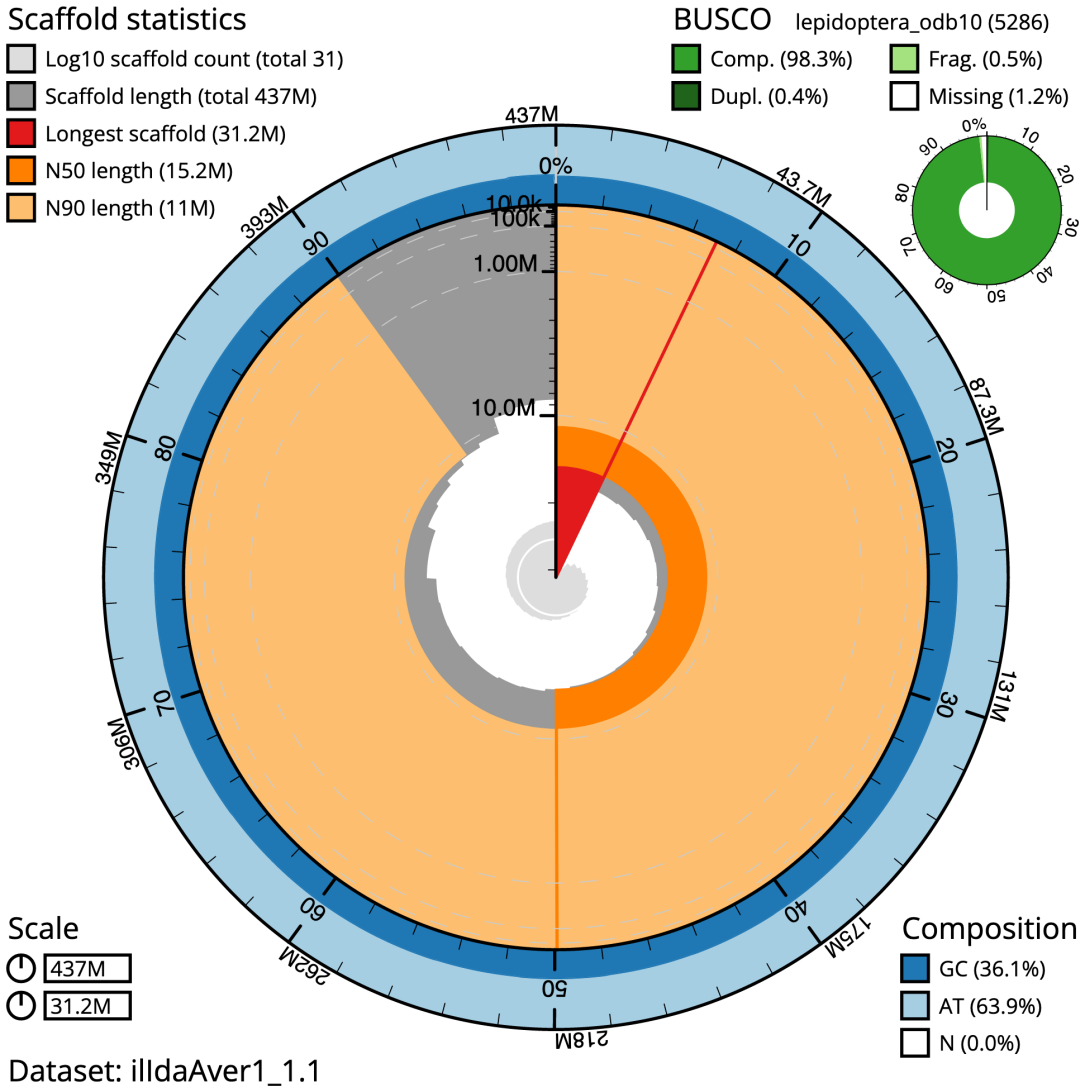
Genome assembly of *Idaea aversata*, ilIdaAver1.1: metrics. The BlobToolKit Snailplot shows N50 metrics and BUSCO gene completeness. The main plot is divided into 1,000 size-ordered bins around the circumference with each bin representing 0.1% of the 436,730,885 bp assembly. The distribution of chromosome lengths is shown in dark grey with the plot radius scaled to the longest chromosome present in the assembly (31,234,832 bp, shown in red). Orange and pale-orange arcs show the N50 and N90 chromosome lengths (15,151,489 and 11,000,729 bp), respectively. The pale grey spiral shows the cumulative chromosome count on a log scale with white scale lines showing successive orders of magnitude. The blue and pale-blue area around the outside of the plot shows the distribution of GC, AT and N percentages in the same bins as the inner plot. A summary of complete, fragmented, duplicated and missing BUSCO genes in the lepidoptera_odb10 set is shown in the top right. An interactive version of this figure is available at https://blobtoolkit.genomehubs.org/view/ilIdaAver1.1/dataset/ilIdaAver1_1.1/snail.

The final assembly has a total length of 436.7 Mb in 30 sequence scaffolds with a scaffold N50 of 15.2 Mb ( Table 1). The whole assembly sequence was assigned to 30 chromosomal-level scaffolds, representing 29 autosomes and the Z sex chromosome. Chromosome-scale scaffolds confirmed by the Hi-C data are named in order of size ( Figure 2– Figure 5; Table 2). The assembly has a BUSCO v5.3.2 ( Manni *et al.*, 2021) completeness of 98.3% (single 97.9%, duplicated 0.4%) using the lepidoptera_odb10 reference set ( *n* = 5,286). While not fully phased, the assembly deposited is of one haplotype. Contigs corresponding to the second haplotype have also been deposited.

**Table 1.** Genome data for *Idaea aversata*, ilIdaAver1.1.

Project accession data		
Assembly identifier	ilIdaAver1.1	
Species	*Idaea aversata*	
Specimen	ilIdaAver1	
NCBI taxonomy ID	104447	
BioProject	PRJEB44980	
BioSample ID	SAMEA7519834	
Isolate information		
Assembly metrics *		*Benchmark*
Consensus quality (QV)	58.3	*≥ 50*
*k*-mer completeness	100%	*≥ 95%*
BUSCO **	C:98.3%[S:97.9%,D:0.4%], F:0.5%,M:1.2%,n:5,286	*C ≥ 95%*
Percentage of assembly mapped to chromosomes	100%	*≥ 95%*
Sex chromosomes	Z chromosome assembled	*localised homologous pairs*
Organelles	Mitochondrial genome assembled	*complete single alleles*
Raw data accessions		
PacificBiosciences SEQUEL II	ERR6565942	
10X Genomics Illumina	ERR6054727–ERR6054730	
Hi-C Illumina	ERR6054731	
Genome assembly		
Assembly accession	GCA_907269075.1	
*Accession of alternate haplotype*	GCA_907269045.1	
Span (Mb)	436.7	
Number of contigs	36	
Contig N50 length (Mb)	14.9	
Number of scaffolds	30	
Scaffold N50 length (Mb)	15.2	
Longest scaffold (Mb)	31.2	
Genome annotation		
Number of protein-coding genes	10,165	
Number of non-coding genes	962	
Average length of coding sequence (bp)	1,502.47	
Average number of exons per transcript	7.08	
Average number of introns per transcript	6.08	
Average intron length (bp)	1,676.92	

* Assembly metric benchmarks are adapted from column VGP-2020 of “Table 1: Proposed standards and metrics for defining genome assembly quality” from ( Rhie *et al.*, 2021). ** BUSCO scores based on the lepidoptera_odb10 BUSCO set using v5.3.2 C = complete [S = single copy, D = duplicated], F = fragmented, M = missing, n = number of orthologues in comparison. A full set of BUSCO scores is available at https://blobtoolkit.genomehubs.org/view/ilIdaAver1.1/dataset/ilIdaAver1_1.1/busco.

**Figure 3.**
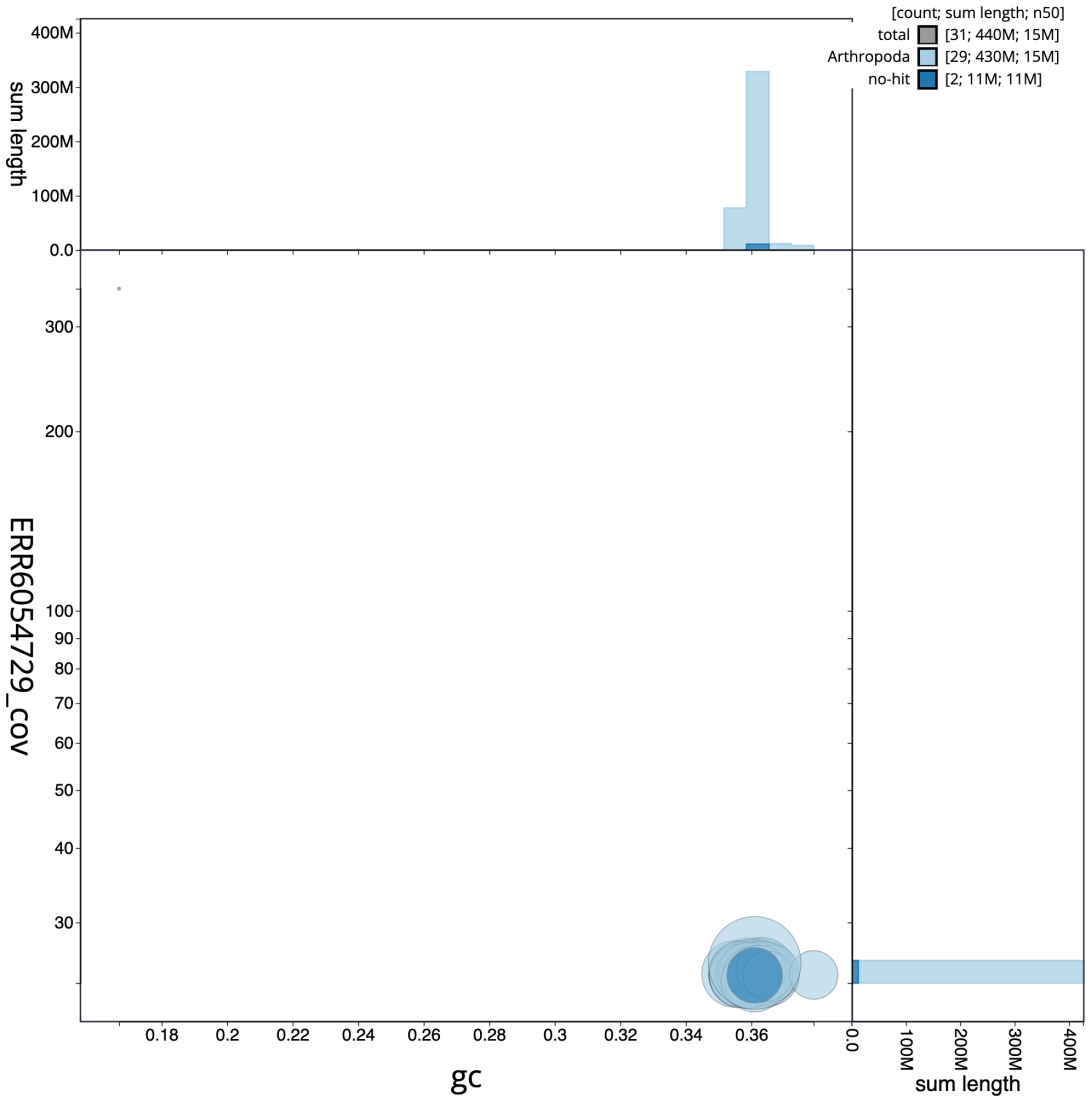
Genome assembly of *Idaea aversata*, ilIdaAver1.1: GC coverage. BlobToolKit GC-coverage plot. Scaffolds are coloured by phylum. Circles are sized in proportion to scaffold length. Histograms show the distribution of scaffold length sum along each axis. An interactive version of this figure is available at https://blobtoolkit.genomehubs.org/view/ilIdaAver1.1/dataset/ilIdaAver1_1.1/blob.

**Figure 4.**
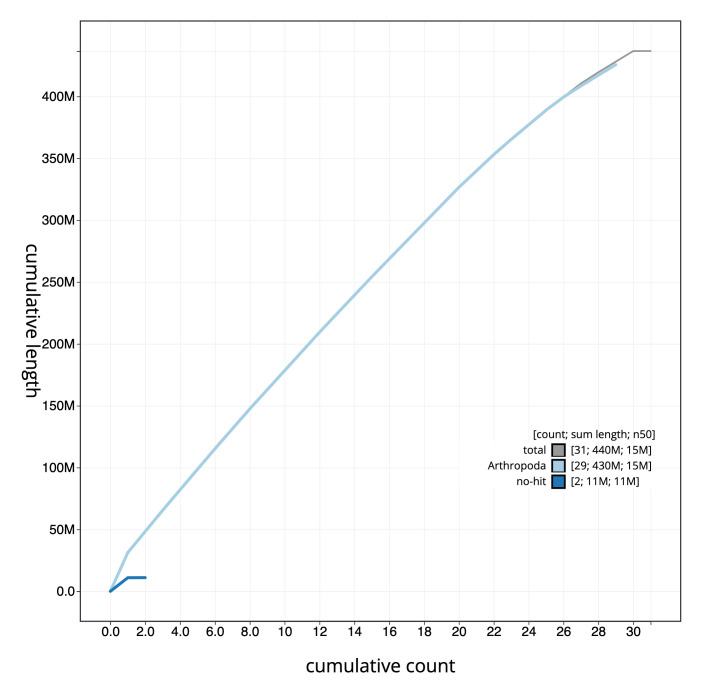
Genome assembly of *Idaea aversata*, ilIdaAver1.1: cumulative sequence. BlobToolKit cumulative sequence plot. The grey line shows cumulative length for all scaffolds. Coloured lines show cumulative lengths of scaffolds assigned to each phylum using the buscogenes taxrule. An interactive version of this figure is available at https://blobtoolkit.genomehubs.org/view/ilIdaAver1.1/dataset/ilIdaAver1_1.1/cumulative.

**Figure 5.**
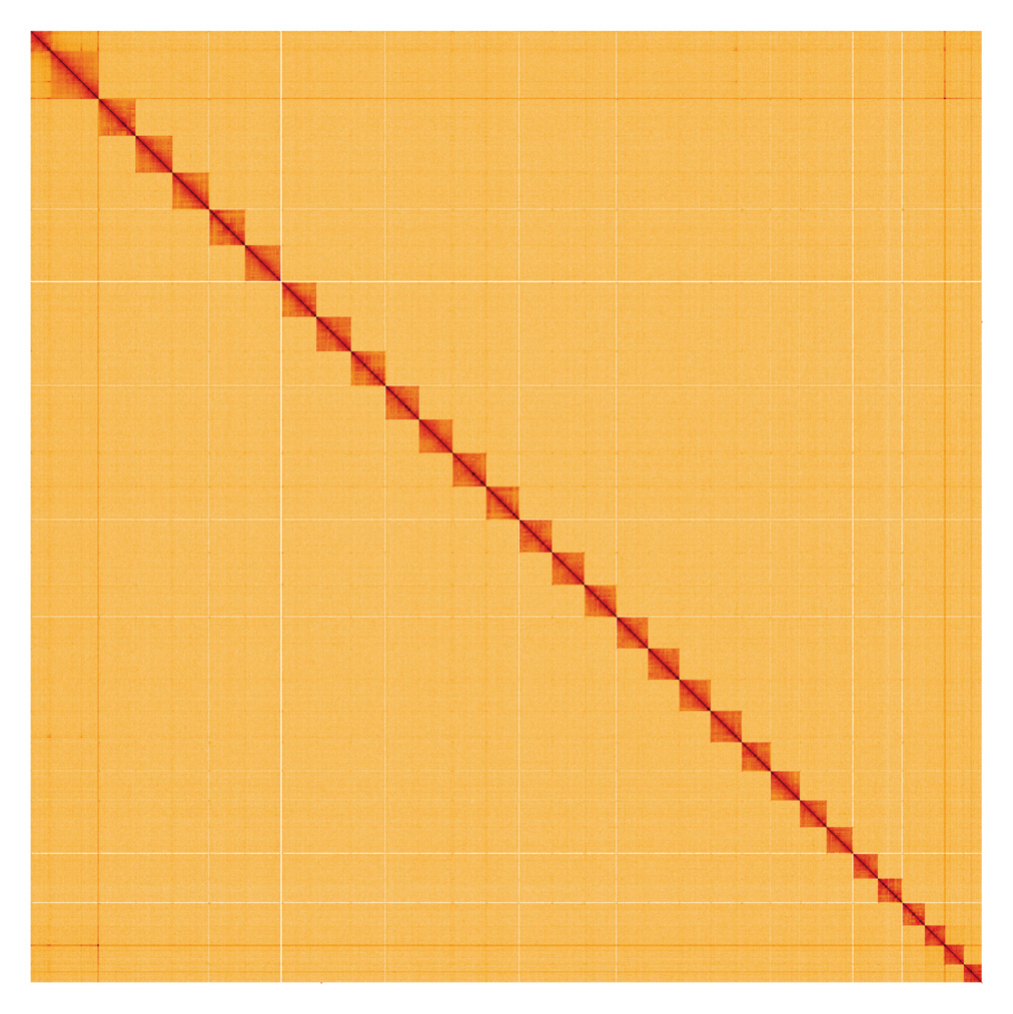
Genome assembly of *Idaea aversata*, ilIdaAver1.1: Hi-C contact map. Hi-C contact map of the ilIdaAver1.1 assembly, visualised using HiGlass. Chromosomes are shown in order of size from left to right and top to bottom. An interactive version of this figure may be viewed at https://genome-note-higlass.tol.sanger.ac.uk/l/?d=IgaKGHGXTsqhcb8DTlA-Mw.

**Table 2.** Chromosomal pseudomolecules in the genome assembly of *Idaea aversata*, ilIdaAver1.

INSDC accession	Chromosome	Size (Mb)	GC%
OU026084.1	1	17.19	36.3
OU026085.1	2	17.03	36.1
OU026086.1	3	16.76	36.3
OU026087.1	4	16.53	36.3
OU026088.1	5	16.45	35.9
OU026089.1	6	16.28	35.9
OU026090.1	7	15.97	35.5
OU026091.1	8	15.69	35.7
OU026092.1	9	15.48	35.7
OU026093.1	10	15.39	36
OU026094.1	11	15.37	35.7
OU026095.1	12	15.15	36.2
OU026096.1	13	14.9	35.7
OU026097.1	14	14.81	36.1
OU026098.1	15	14.63	36
OU026099.1	16	14.61	36
OU026100.1	17	14.42	36.5
OU026101.1	18	14.39	36.3
OU026102.1	19	14.28	35.9
OU026103.1	20	13.22	36.1
OU026104.1	21	13.22	36.2
OU026105.1	22	12.32	36
OU026106.1	23	12.04	36.1
OU026107.1	24	11.52	36.6
OU026108.1	25	11	36.1
OU026109.1	26	10.6	35.9
OU026110.1	27	9.06	36.3
OU026111.1	28	8.72	36.5
OU026112.1	29	8.45	37.9
OU026083.1	Z	31.23	36.1
OU026113.1	MT	0.02	16.9

## Genome annotation report

Annotation of the GCA_907269075.1 assembly was generated using the Ensembl genome annotation pipeline. The resulting annotation includes 10165 protein coding genes with an average length of 12352.21 and an average coding length of 1502.47, and 962 non-protein coding genes. There is an average of 7.08 exons and 6.08 introns per canonical protein coding transcript, with an average intron length of 1676.92. A total of 4000 gene loci have more than one associated transcript. The annotation identified a repeat content of 44.89%.

## Methods

### Sample acquisition and nucleic acid extraction

A male *Idaea aversata* (ilIdaAver1) was collected from Wytham Woods, Oxfordshire (biological vice-county: Berkshire), UK (latitude 51.77, longitude –1.34) on 29 June 2019 using a light trap. The specimen was collected and identified by Douglas Boyes (University of Oxford) and snap-frozen on dry ice by Peter Holland. The specimen is of the unbanded form *remutata*.

DNA was extracted at the Tree of Life laboratory, Wellcome Sanger Institute (WSI). The ilIdaAver1 sample was weighed and dissected on dry ice with tissue set aside for Hi-C sequencing. Whole organism tissue was disrupted using a Nippi Powermasher fitted with a BioMasher pestle. High molecular weight (HMW) DNA was extracted using the Qiagen MagAttract HMW DNA extraction kit. Low molecular weight DNA was removed from a 20 ng aliquot of extracted DNA using 0.8X AMpure XP purification kit prior to 10X Chromium sequencing; a minimum of 50 ng DNA was submitted for 10X sequencing. HMW DNA was sheared into an average fragment size of 12–20 kb in a Megaruptor 3 system with speed setting 30. Sheared DNA was purified by solid-phase reversible immobilisation using AMPure PB beads with a 1.8X ratio of beads to sample to remove the shorter fragments and concentrate the DNA sample. The concentration of the sheared and purified DNA was assessed using a Nanodrop spectrophotometer and Qubit Fluorometer and Qubit dsDNA High Sensitivity Assay kit. Fragment size distribution was evaluated by running the sample on the FemtoPulse system.

### Sequencing

Pacific Biosciences HiFi circular consensus and 10X Genomics read cloud DNA sequencing libraries were constructed according to the manufacturers’ instructions. DNA sequencing was performed by the Scientific Operations core at the WSI on Pacific Biosciences SEQUEL II (HiFi) and HiSeq X Ten (10X) instruments. Hi-C data were also generated from tissue of ilIdaAver1 using the Arima v2 kit and sequenced on the Illumina NovaSeq 6000 instrument.

### Genome assembly

Assembly was carried out with Hifiasm ( Cheng *et al.*, 2021) and haplotypic duplication was identified and removed with purge_dups ( Guan *et al.*, 2020). One round of polishing was performed by aligning 10X Genomics read data to the assembly with Long Ranger ALIGN, calling variants with freebayes ( Garrison & Marth, 2012). The assembly was then scaffolded with Hi-C data ( Rao *et al.*, 2014) using SALSA2 ( Ghurye *et al.*, 2019). The assembly was checked for contamination and corrected using the gEVAL system ( Chow *et al.*, 2016) as described previously ( Howe *et al.*, 2021). Manual curation was performed using gEVAL, HiGlass ( Kerpedjiev *et al.*, 2018) and Pretext ( Harry, 2022). The mitochondrial genome was assembled using MitoHiFi ( Uliano-Silva *et al.*, 2022), which performed annotation using MitoFinder ( Allio *et al.*, 2020). The genome was analysed and BUSCO scores generated within the BlobToolKit environment ( Challis *et al.*, 2020). Table 3 contains a list of all software tool versions used, where appropriate.

**Table 3.** Software tools and versions used.

Software tool	Version	Source
BlobToolKit	3.5.2	Challis *et al.*, 2020
freebayes	1.3.1-17-gaa2ace8	Garrison & Marth, 2012
gEVAL	N/A	Chow *et al.*, 2016
Hifiasm	0.12	Cheng *et al.*, 2021
HiGlass	1.11.6	Kerpedjiev *et al.*, 2018
Long Ranger ALIGN	2.2.2	https://support.10xgenomics.com/genome-exome/software/pipelines/latest/advanced/other-pipelines
MitoHiFi	1	Uliano-Silva *et al.*, 2022
PretextView	0.2	Harry, 2022
purge_dups	1.2.3	Guan *et al.*, 2020
SALSA	2.2	Ghurye *et al.*, 2019

### Genome annotation

The Ensembl gene annotation system ( Aken *et al.*, 2016) was used to generate annotation for the *I. aversata* assembly (GCA_907269075.1). Annotation was created primarily through alignment of transcriptomic data to the genome, with gap filling via protein to-genome alignments of a select set of proteins from UniProt ( UniProt Consortium, 2019).

### Ethics/compliance issues

The materials that have contributed to this genome note have been supplied by a Darwin Tree of Life Partner. The submission of materials by a Darwin Tree of Life Partner is subject to the Darwin Tree of Life Project Sampling Code of Practice. By agreeing with and signing up to the Sampling Code of Practice, the Darwin Tree of Life Partner agrees they will meet the legal and ethical requirements and standards set out within this document in respect of all samples acquired for, and supplied to, the Darwin Tree of Life Project. Each transfer of samples is further undertaken according to a Research Collaboration Agreement or Material Transfer Agreement entered into by the Darwin Tree of Life Partner, Genome Research Limited (operating as the Wellcome Sanger Institute), and in some circumstances other Darwin Tree of Life collaborators.

## Data Availability

European Nucleotide Archive: *Idaea aversata* (riband wave). Accession number PRJEB44980; https://identifiers.org/ena.embl/PRJEB44980 ( Wellcome Sanger Institute, 2022). The genome sequence is released openly for reuse. The *Idaea aversata* genome sequencing initiative is part of the Darwin Tree of Life (DToL) project. All raw sequence data and the assembly have been deposited in INSDC databases. Raw data and assembly accession identifiers are reported in Table 1.

## Author information

Members of the University of Oxford and Wytham Woods Genome Acquisition Lab are listed here: https://doi.org/10.5281/zenodo.4789928.

Members of the Darwin Tree of Life Barcoding collective are listed here: https://doi.org/10.5281/zenodo.4893703.

Members of the Wellcome Sanger Institute Tree of Life programme are listed here: https://doi.org/10.5281/zenodo.4783585.

Members of Wellcome Sanger Institute Scientific Operations: DNA Pipelines collective are listed here: https://doi.org/10.5281/zenodo.4790455.

Members of the Tree of Life Core Informatics collective are listed here: https://doi.org/10.5281/zenodo.5013541.

Members of the Darwin Tree of Life Consortium are listed here: https://doi.org/10.5281/zenodo.4783558.
