# Supplementary material for: The genome sequence of the Riband Wave, Idaea aversata (Linnaeus, 1758)
Source: Wellcome Open Res. 2023 Jan 31;8:45. [Version 1] doi: 10.12688/wellcomeopenres.18899.1 (PMC10357074; doi:10.12688/wellcomeopenres.18899.1)
Supplement: Peer review history

# Peer review: Reviewer response for version 1

**DOI:** 10.21956/wellcomeopenres.20956.r62860

**Published:** 2023-07-19

**Authors:** Amanda Markee

**Affiliations:** 1 McGuire Center for Lepidoptera and Biodiversity, University of Florida, Gainesville, Florida, USA

The authors here provide a well documented chromosome level assembly for *Idaea aversata, *a geometrid moth found throughout the UK. This species has unique genotypes related to wing pattern variation, making the full genome useful for studying color polymorphism in Lepidoptera. The methods outlined for isolating nucleic acids were clear and reproducible, I would however add catalog numbers for each kit that was mentioned, if this is in line with the format for this journal. Sequencing methods and materials were clearly explained, and downstream bioinformatic methods were written in chronology of the workflow, making it easy to follow. In the genome sequence report section, similar to the genome annotation report section, I recommend summarizing more information from the figures, including other genome metrics like L50, BlobPlot results, and GC content. Data availability and reuse potential are clearly outlined in the manuscript. My last recommendation is to clarify which select set of proteins were used from UniProt database for the protein annotation (i.e. from insects, arthropods, or geometrids specifically).

Are sufficient details of methods and materials provided to allow replication by others?

Yes

Is the rationale for creating the dataset(s) clearly described?

Yes

Are the datasets clearly presented in a useable and accessible format?

Yes

Are the protocols appropriate and is the work technically sound?

Yes

Reviewer Expertise:

Lepidoptera, genomics, silk evolution

I confirm that I have read this submission and believe that I have an appropriate level of expertise to confirm that it is of an acceptable scientific standard.

# Peer review: Reviewer response for version 1

**DOI:** 10.21956/wellcomeopenres.20956.r54594

**Published:** 2023-02-03

**Authors:** Sean T. S. Law

**Affiliations:** 1 School of Life Sciences, Simon F.S. Li Marine Science Laboratory, State Key Laboratory of Agrobiotechnology, The Chinese University of Hong Kong, Hong Kong, China

This article presents both the nuclear and mitochondrial genomes of a male Riband Wave *Idaea aversata *(Linnaeus, 1758), particularly of the unbanded form. It is intriguing that the banded pattern is known to be determined by a dominant allele, which varies in frequency in different localities according to previous records. The colour polymorphism of this species also raised interesting biological questions.

 The *I. aversata *genome is assembled with cutting-edge tools and standards. This resulted in high completeness (BUSCO score 98.3%) and contiguity (N50 at ~15Mb) with majority of scaffolds anchored into 30 chromosomes including the Z sex chromosome. While most of the genome statistics and methods are well summarised and described, it is recommended to consider further description on how the Z chromosome was identified with “localised homologous pairs” (Table 1) in the methods section (e.g., what alignment/synteny tools were used? Which organism(s) were compared?). Nevertheless, the article is well-written and concise overall. The high-quality genome of *I. aversata *in this study provides useful genomic resources for further investigations in this species and other lepidopterans.

Are sufficient details of methods and materials provided to allow replication by others?

Yes

Is the rationale for creating the dataset(s) clearly described?

Yes

Are the datasets clearly presented in a useable and accessible format?

Yes

Are the protocols appropriate and is the work technically sound?

Yes

Reviewer Expertise:

Evolutionary biology, population genomics

I confirm that I have read this submission and believe that I have an appropriate level of expertise to confirm that it is of an acceptable scientific standard.
